# A new terpene coumarin microbial transformed by *Mucor polymorphosporus* induces apoptosis of human gastric cancer cell line MGC-803

**DOI:** 10.1007/s12272-018-1028-0

**Published:** 2018-04-04

**Authors:** Guangzhi Li, Junchi Wang, Xiaojin Li, Jianguo Xu, Zhao Zhang, Jianyong Si

**Affiliations:** 1 Key Laboratory of Bioactive Substances and Resources Utilization of Chinese Herbal Medicine, Ministry of Education, Institute of Medicinal Plant Development, Peking Union Medical College, Chinese Academy of Medical Sciences, Beijing, 100193 People’s Republic of China; 2 Shenzhen Luohu People’s Hospital, The Third Affiliated Hospital of Shenzhen University, Shenzhen, 518035 People’s Republic of China; 3 Xinjiang Institute of Chinese Materia Medica and Ethical Materia Medica, Ürümqi, 830002 People’s Republic of China

**Keywords:** Biotransformation, *Mucor polymorphosporus*, Terpene coumarin, Human gastric cancer MGC-803 cell line, Apoptosis

## Abstract

2′-Z auraptene (**1**) is a synthesized monoterpene coumarin with anticancer activity against human gastric cancer cells. In order to find new potential anticancer agent, *Mucor polymorphosporus* was used to transform *cis*-auraptene. Four new terpene coumarins with notable changes in the skeletal backbone, 2′-Z auraptene A-D (**2**–**5**), were obtained and evaluated for their antiproliferative effects against human normal gastric epithelium cells and human gastric cancer cells. These new compounds showed selective cytotoxic activity against MGC-803 cells with IC_50_ values from 0.78 ± 0.13 to 10.78 ± 1.83 μM and the therapeutic index could also be significantly improved (TI = 59.0) compared with that of **1** (TI = 5.5). The structures of these metabolites were elucidated through extensive spectroscopic methods, and the possible biotransformation pathway of **1** by *Mucor polymorphosporus* was also proposed. Furthermore, the mechanism of the antiproliferative effects against MGC-803 cells of the most potent compound, 2′-Z auraptene A (**2**), was characterized. Annexin V/PI staining and abnormal expression of apoptosis-related protein suggested that compound **2** induces apoptosis in gastric cancer MGC-803 cells. Therefore, it is possible that compound **2** has the potential to be applied in gastric cancer therapy.

**Electronic supplementary material:**

The online version of this article (10.1007/s12272-018-1028-0) contains supplementary material, which is available to authorized users.

## Introduction

Gastric cancer is one of the most common malignancies in the world and characterized by its high mortality rate (Yu et al. 2014). It was estimated that nearly 700,000 people die from it and approximately 1,000,000 new patients are diagnosed worldwide each year (Parkin et al. 2005). In spite of great attention has been paid on the treatment of gastric cancer and a great improvement has emerged in the treatment of this disease, but the median survival time for patients with this disease is still only 6–9 months (Verdecchia et al. 2004). Currently, chemotherapy is a mainly approach for the treatment of gastric cancer (Grant 2009), and drug resistance is one of the most significant obstacles in chemotherapy. Besides, toxicities of using high doses of chemotherapeutic agents to get over drug resistance also significantly impede patients′ recovery (Vijayaraghavalu et al. 2012; Solyanik 2010). Therefore, it is urgent and necessary to find novel anticancer agents with potent activity and high therapeutic index to improve current treatment of gastric cancer patients (Riedl et al. 2011).

**Fig. 1:**
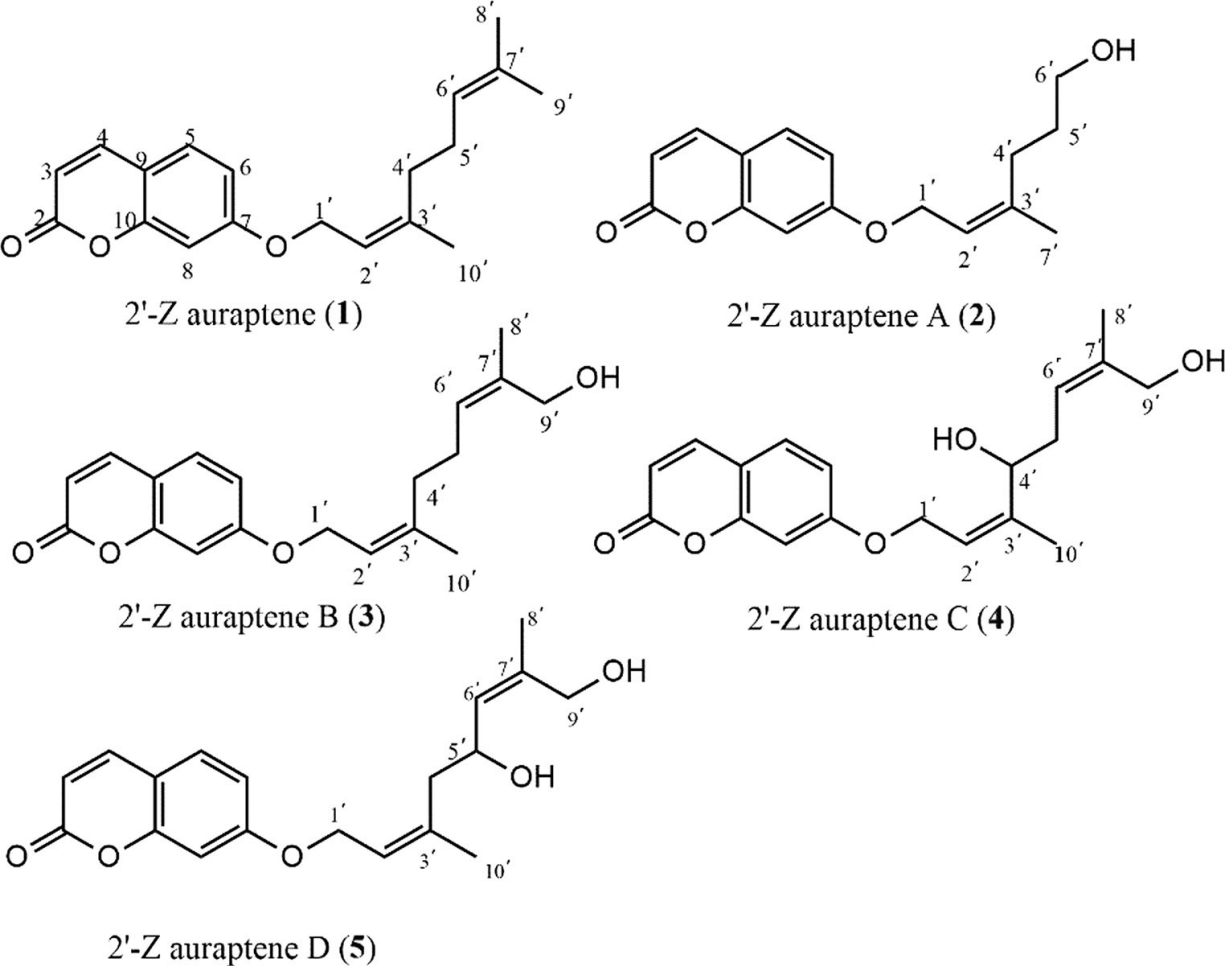
Structures of compounds **1**–**5**

Coumarins are a wide family of natural and synthetic compounds, which exhibited a wide range of pharmacological activities including antitumor (Barthomeuf et al. 2008), antioxidant (Kostova et al. 2011), anti-inflammatory (Curini et al. 2004), antibacterial and antiviral (Massimo et al. 2003) activities. Based on previously numerous researches, 7-prenyloxycoumarins have been described as agents for cancer chemoprevention including the treatment for gastric cancer (Gliszczyńska and Brodelius 2012; Zhang et al. 2015b). 2′-Z auraptene (Fig. 1), a member of 7-prenyloxycoumarins, was synthesized in our research according to previous study (Coates and Melvin 1970), which exhibits anticancer activity against gastric cancer cells (IC_50_ = 10.38 ± 0.86 μM, TI = 5.5).

Biotransformation has been widely employed in the structural modification of organic compounds to find more promising lead compounds in the development of new drugs. The whole cell, dead microorganism or enzymes all could act as catalyst, which catalyze kinds of chemical reactions, such as oxidation, reduction and hydroxylation (Smith et al. 2015). In addition, compared with traditional chemical modification, microbial transformation has numerous superiorities, such as higher stereo- and regioselectivity, higher efficiency, lower pollution, and less cost (Dong et al. 2016). Mucorales is the largest family of the Zygomycetes, which is applied extensively in biotechnology for organic acid and enzyme production. *Mucor polymorphosporus*, an impotant member of Mucorales, has been commonly employed as catalysts to find the new drug candidates in the structural modification of terpenoid (Silva et al. 2013). In the present study, we investigated the biotransformation of **1** by *Mucor polymorphosporus*, four new terpene coumarins 2′-Z auraptene A (**2**), B (**3**), C (**4**), D (**5**) (Fig. 1) were obtained and screened subsequently for their antiproliferative effects against gastric cancer MGC-803 cell line. We found that compound **2** was the most potent against gastric cancer cells with the least cytotoxic to normal gastric epithelial cells among these compounds and could induce caspase-dependent apoptosis in gastric cancer MGC-803 cells.

## Materials and methods

### Materials

Pre-coated silica gel plates (Zhi Fu Huang Wu Pilot Plant of Silica Gel Development, Yantai, P. R. China) were used to perform thin layer chromatography analysis. Sephadex LH-20 (Pharmacia; Uppsala, Sweden) and silica gel (Yantai Oceanic Chemicals, Yantai, P. R. China) were used for column chromatography. Semi-preparative HPLC was performed on a K1001 analytic LC instrument (Beijing Chuangxintongheng Science & Technology Co., Ltd,) equipped with a K-2600 UV detector and a YMC-Pack ODS-A column (250 × 10 mm, S-5 µm, 12 nm). Nuclear magnetic resonance (NMR) spectra were recorded using Bruker AVANCE Ш 600 spectrometer (Bruker BioSpin, Rheinstetten, German) operating at 150 MHz for ^13^C and at 600 MHz for 1H). High resolution electrospray ionization mass spectrometry (HR–ESI–MS) was performed on a Thermo Scientific LTQ-Obitrap XL (Thermo Fisher Scientific; Bremen, Germany). Phosphate Buffered Saline (PBS) and Dulbecco′s modified Eagle′s medium (DMEM) were provided by HyClone (CA, San Jose, USA). Fetal bovine serum (FBS), penicillin, streptomycin, and Trypsin–EDTA were purchased from Solarbio (Beijing, China). DMSO (cell culture grade) and 3-(4, 5-dimethylthiazol-2-yl)-2, 5-diphenyltetrazolium bromide (MTT) were obtained from Sigma-Aldrich (CA, San Jose, USA). The Annexin V-FITC Apoptosis Kit was supplied by KeyGEN Biotech (Jiangsu, China). The cECL Western Blot Kit was provided by BD Biosciences (CA, San Jose, USA). Antibodies against β-actin, Bax, Bcl-2 and cleaved caspase-3 were obtained from Santa Cruz Biotechnology (CA, San Jose, USA). 7-hydroxycoumarin (≥ 98% purity), nerol (≥ 98% purity), PBr_3_ (≥ 95% purity), pyridine (≥ 98% purity), K_2_CO_3_ (≥ 99% purity), KI (≥ 99.9% purity) were supplied by Energy Chemical (Shanghai, China). All solvents used were obtained from Beijing Chemical Works (Beijing, China).

## The synthesis of 2′-Z auraptene (1)

2′-Z auraptene (**1**) was synthesized as the following methods (Tsangarakis et al. 2007). A mixture of nerol (1.36 g, 10.0 mmol), PBr_3_ (1.35 g, 10.0 mmol), and appropriate pyridine in diethyl ether was placed in ice-water bath with a nitrogen atmosphere. After 0.5 h stirring, ice water was added to terminate reaction, and subsequently the mixture was extracted by diethyl ether to afford the rude intermediation used in the next step without further purification. Then, a mixture of the intermediation obtained from above, 7-hydroxycoumarin (1.62 g, 10.0 mmol), K_2_CO_3_ (1.38 g, 10.0 mmol), and KI (catalytic) in acetone were stirred at room temperature for 2–3 h. Subsequently, the obtained crude mixture was purified by silica gel chromatography, eluting with petroleum ether- ethyl acetate (20:1, v/v).

Compound **1** was obtained as colorless solid (1.19 g, 80% yield) and identified by spectroscopic methods. NMR data of **1** was conform to that of published data for (Z)-7-((3′, 7′-dimethylocta-2′, 6′-dien-1′-yl) oxy)coumarin (Coates and Melvin 1970), so we identified compound **1** as (Z)-7-((3′,7′-dimethylocta-2′,6′-dien-1′-yl)oxy)-2*H*- chromen-2-one, and named it as *cis*-auraptene in our study.

### 2′-Z auraptene A (**2**)

Colorless solid; (+) HR-ESI–MS *m/z* 297.1094 (calcd for C_16_H_18_O_4_Na [M + Na]^+^, 297.1097); $\left[ \alpha \right]^{ 20}_{\text{D}}$-26.7 (*c* 0.086, MeOH); ^1^H NMR data, see Table 1; ^13^C NMR data, see Table 2.

**Table 1:** ^1^H NMR (600 MHz) data for **2**–**5** in CDCl_3_

Position	Compound δ_H_ (J, Hz)			
	2	3	4	5
3	6.24(d, 9.6)	6.24(d, 9.6)	6.25(d, 9.6)	6.25(d, 9.6)
4	7.63(d, 9.6)	7.63(d, 9.6)	7.63(d, 9.6)	7.63(d, 9.6)
5	7.35(d, 9.0)	7.36(d, 9.0)	7.36(d, 9.0)	7.36(d, 8.4)
6	6.84(dd, 9.0, 1.8)	6.85(dd, 8.4, 1.8)	6.85(dd, 8.4, 1.8)	6.84(dd, 8.4, 2.4)
8	6.82(d, 1.8)	6.82(d, 1.8)	6.81(d, 1.8)	6.81(d, 2.4)
1′	4.60(d, 6.6)	4.60(d, 6.6)	4.66(d, 6.6)	4.62(d, 6.6)
2′	5.51(t, 6.6, 12.0)	5.47(t, 6.6, 13.2)	5.74(t, 6.6, 13.2)	5.58(t, 6.6, 13.2)
4′	2.18(t, 7.2, 15.0)	2.21 a	4.14(t, 6.0, 12.6)	2.27 a
5′	1.73 (m)	2.14(m)	2.36(t, 6.6, 13.2)	4.62(m)
6′	3.66(t, 6.6, 13.2)	5.38(t, 6.6, 13.2)	5.42(t, 6.6, 13.2)	5.47(d, 8.4)
7′	1.78(s)			
8′		1.67(s)	1.70(s)	1.73(s)
9′		3.99(s)	4.02(s)	4.02(s)
10′		1.77(s)	1.78(s)	1.83(s)

^a^Overlapping signals

**Table 2:** ^13^C-APT NMR (150 MHz) data for **2**–**5** in CDCl_3_

Position	Compound (δc)			
	2	3	4	5
2	161.45	161.46	161.40	161.40
3	113.39	113.45	113.40	113.40
4	143.60	143.61	143.60	143.60
5	128.85	128.85	128.90	128.90
6	113.17	113.16	113.30	113.30
7	162.21	162.23	162.00	162.10
8	101.69	101.66	101.60	101.60
9	156.01	156.01	156.00	156.00
10	112.62	112.63	112.70	112.70
1′	65.51	65.60	65.20	65.30
2′	118.91	118.92	120.00	122.30
3′	142.16	142.05	142.80	138.70
4′	35.89	39.20	76.40	47.70
5′	30.63	25.76	33.60	66.10
6′	62.66	125.32	120.80	127.30
7′	16.86	135.52	138.40	138.20
8′		13.93	14.20	14.20
9′		69.00	68.70	68.10
10′		16.82	12.90	17.30

^a^Overlapping signals

### 2′-Z auraptene B (**3**)

Colorless solid; (+) HR-ESI–MS *m/z* 337.1406 (calcd for C_19_H_22_O_4_Na [M + Na]^+^, 337.1410); $\left[ \alpha \right]^{ 20}_{\text{D}}$-6.23 (*c* 0.163, MeOH); ^1^H NMR data, see Table 1; ^13^C NMR data, see Table 2.

### 2′-Z auraptene C (**4**)

Colorless solid; (+) HR-ESI–MS *m/z* 353.1355 (calcd for C_19_H_22_O_5_Na [M + Na]^+^, 353.1359); $\left[ \alpha \right]^{ 20}_{\text{D}}$-21.04 (*c* 0.243, MeOH); ^1^H NMR data, see Table 1; ^13^C NMR data, see Table 2.

### 2′-Z auraptene D (**5**)

Colorless solid; (+) HR-ESI–MS *m/z* 353.1356 (calcd for C_19_H_22_O_5_Na [M + Na]^+^, 353.1359); $\left[ \alpha \right]^{ 20}_{\text{D}}$-17.16 (*c* 0.083, MeOH); ^1^H NMR data, see Table 1; ^13^C NMR data, see Table 2.

## Microorganism and culture medium

The fugal strain, *Mucor polymorphosporus* (*CGMC3.3443*), was obtained from the China General Microbiological Culture Collection Center. *Mucor polymorphosporus* was cultured on potato dextrose agar slants at 37 °C and stored at 4 °C. The whole biotransformation process were performed in potato medium, which was prepared according to the following methods: A mixture of distilled water (1000 mL) and mincing husked potato (250 g) was boiled for half an hour, and then was filtered. Subsequently, the filtrate was mixture with distilled water and glucose (20.0 g), and was autoclaved at 121 °C half an hour.

### Biotransformation

The strain screening experiment was performed as follows: Fungus mycelia were seeded in 1000-mL erlenmeyer flasks containing 500 mL culture medium, and cultured in a rotary shaker for 2 days (37 °C, 160 rpm). Then, compound **1** (500.0 mg) dissolved in methanol was added to each flask with another 4-day incubation. Finally, the broth was extracted by an equal volume of ethyl acetate for three times, and the organic solvent was vacuum dried to obtain a rude mixture (Gao et al. 2017).

### Isolation of metabolites

The crude extract (1.1 g), eluted with petroleum ether-ethyl acetate (20:1 to 0:1, v/v), was fractionated into nine fractions (Fr. 1–9) by chromatography using a silica gel column (200–300 mesh, 25.0 g). These fractions were separated by semi-preparative HPLC eluted with MeOH–H_2_O on a Thermo C18 column (S-5 µm, 12 nm, 250 × 10 mm), respectively. Finally, compound **2** (35.6 mg, t_R_ 18.6 min) was yielded from Fr. 2 eluting with MeOH–H_2_O (64:36) system; Compound **3** (21.3 mg, t_R_ 24.2 min) was obtained from Fr. 3 eluted with MeOH–H_2_O (65:35) system; Compound **4** (18.6 mg, t_R_ 17.5 min) was separated from Fr. 5 using a MeOH–H_2_O (57:48) system; Compound **5** (35.6 mg, t_R_ 16.8 min) was separated from Fr. 6 eluting with a MeOH–H_2_O (55:45) system.

### Cell culture

The human gastric cancer cell line MGC-803 and human normal gastric epithelial cell line GES-1 were cultured in DMEM containing penicillin (100 U/mL), streptomycin (100 μg/mL), and 10% FBS at 37 °C with 5% CO_2_. Cells that used in experiments were passaged at least three times.

### Antiproliferation effect assays

Cells were seeded in 96-well plates at a density of 5 × 10^4^/well and cultured at 37 °C with 5% CO_2_ for 24 h. And then, compounds (**1**–**5**) in various concentrations were introduced with incubation for another 48 h. Finally, 10 μL MTT (5 mg/mL) was added to each well. After 4 h incubation, the medium was removed and 150 μL DMSO was added. Absorbance was read on a Microplate Reader at 570 nm (Bio Tek, Thenceforth, USA) (Li et al. 2014). The MTT assay aims to evaluate the antiproliferative effects of the test compounds in MGC-803 cells and the cytotoxic effects in GES-1 cells.

### FACS analysis

The apoptosis effect of 2′-Z auraptene A (**2**) in MGC-803 cells was examined using the Annexin V-FITC/PI detection kit, according to the description of previously reported (Bakar et al. 2016). Briefly, cells were seeded and treated with compound **2** (0, 1.0, 2.0, 4.0 μM) for 24 h. Cells were harvested and washed twice with cold PBS before incubating with annexin V and PI for 30 min in dark. And then the stained cells were detected and analyzed on FACS Calibur flow cytometry.

### Western blot

The detection of levels of apotosis-related proteins in MGC-803 cells was performed according to the reported western blot method (Zhang et al. 2015a). Briefly, cells were treated with compound **2** (0, 1.0, 2.0, 4.0 μM) for 24 h and then harvested to detect the protein concentration using the BCA method. Total cellular protein was separated by an SDS/PAGE gel and transferred onto PVDF membranes using a semidry electroblotter. The membrane was blocked with non-fat milk solution (5%) for 1 h, and incubated subsequently with primary antibody at 4 °C overnight. Then TBST was used to wash the primary antibody and incubated with secondary antibody for 1 h at room temperature. Protein bands were visualized by enhanced chemiluminescence (ECL) and β-actin was used as a normalizing control.

### Statistical analysis

Datas are expressed as mean ± SD of three independent experiments. IBM SPSS statistics 19 was used for data analysis. Statistical significant differences were established at p < 0.05.

## Results and discussion

### The structure elucidation of new compounds

2′-Z auraptene A (**2**) was obtained as colorless solid with a molecular formula of C_16_H_18_O_4_ determined from (+) HR-ESI–MS *m/z* 297.1094 (calcd for C_16_H_18_O_4_Na [M + Na]^+^, 297.1097). The NMR data of compound **2** (Table 1 and Table 2) in downfield region were almost same with that of compound **1**, which indicated the existence of the structure of 7-*O*-substituted coumarin in **2**. The ^13^C-APT NMR spectrum of **2** exhibited one methyl group signal at δc 16.86, one trisubstituted olefinic bond signals at δc 118.91, 142.16, two primary oxygenated carbon signals at δc 65.51, 62.66 and two aliphatic methylene group signals at δc 35.89, 30.63. In the ^1^H NMR spectrum of **2**, the signals at 1.73 (s), 4.60 (d, 6.6 Hz) and 5.51 (t, 6.6, 12.0) indicated the presence of the partial structure of C-1′-C-2-C3′-C-7′, which was same with that of **1**. Besides, considering the coupling relationships of the remaining three carbons, we confirmed the existence of the partial structure of C-3′-C-4′-C-5′. Therefore, we identified compound **2** as (Z)-7-((3′,7′-dimethylocta-2′,6′-dien-1′-yl) oxy)- 2*H*-chromen-2-one and named it as 2′-Z auraptene A.

**Fig. 2:**
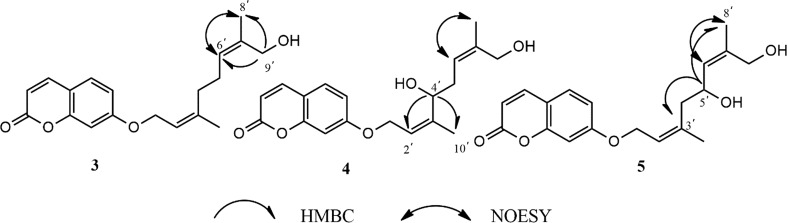
The 2D NMR data of compounds **3**–**5**

2′-Z auraptene B (**3**) possessed a molecular formula of C_19_H_22_O_4_ determined from ^13^C-APT NMR data and (+) HR-ESI–MS ion peak at *m/z* 337.1406 (calcd for C_19_H_22_O_4_Na [M + Na]^+^, 337.1410). The NMR data of **3** (Table 1-2) were similar with those of **1**, except that the methyl signals (δ_H_ 1.74, δc 15.36) in **1** were replaced by the primary oxygenated methylene signals (δ_H_ 3.99, δc 69.00). The substituted position of the primary oxygenated methylene in **3** was determined by the HMBC correlations from H-9′(3.99, 69.00) to C-6′ and C-8′ (Fig. 2). Besides, in the NOESY spectrum, the correlations between H-6′(δ_H_ 5.38) and H-8′(δ_H_ 1.67) confirmed the configuration of C-6′ was Z. Finally, we established the new compound as 7-(((2′Z,6′Z)-9′-hydroxy-3′,7′-dimethylocta-2′,6′-dien-1′-yl)oxy)-2*H*-chromen-2- one and we named it as 2′-Z auraptene B.

2′-Z auraptene C (**4**) was isolated as colorless solid. Its molecular formula was deduced as C_19_H_22_O_5_ on the basis of ^13^C-APT NMR data and (+) HRESIMS ion peak at *m/z* 353.1355 (calcd for C_19_H_22_O_5_Na [M + Na]^+^, 353.1359). The NMR data of **4** (Table 1-2) were similar with those of **3**, except that the aliphatic methylene group signals at δ_H_ 2.21, δ_c_ 39.20 of **3** were replaced by oxygenated methine signals at δ_H_ 4.14, δ_c_ 76.40. The substituted position of the oxygenated methine in **4** was determined by the HMBC correlations from H-4′ to C-2′ and C-10′ (Fig. 2). The NOESY correlation signals of H-6′ (δ_H_ 5.42) and H-8′ (δ_H_ 1.70) were also observed in compound **4**. Overall, we identified the new compound as 7-(((2′Z,6′Z)-4′,9′-hydroxy-3′,7′-dimethylocta-2′,6′-dien-1′-yl)oxy)-2*H*-chromen-2-one and we named it as 2′-Z auraptene C.

2′-Z auraptene D (**5**) was obtained as colorless solid. The molecular formula was established from ^13^C-APT NMR data and (+) HRESIMS ion peak at *m/z* 353.1356 (calcd for C_19_H_22_O_5_Na [M + Na]^+^, 353.1359). The NMR data of **4** almostly same with those of **5** (Tables 1, 2), but the chemical shift of C-3′ (δ_c_ 142.80) shifted to high field region (δ_c_ 138.70) and C-6′ (δ_c_ 120.80) shifted to down field region (δ_c_ 127.30) reversely. Considering the significantly influence of the substituted position of C-4′ and C-5′ to C-3′ and C-6′, the position of the hydroxyl in **4** must be different from that of compound **5**. In the HMBC spectrum, the correlations from H-5′ (δ_H_ 4.62) to C-3′ (δ_c_ 138.70) and C-8′ (δ_c_ 14.20) (Fig. 2) confirmed that the position of the hydroxyl group at C-5′ in compound **5**. Besides, there was also correlation signals between H-6′(δ_H_ 5.47) and H-8′(δ_H_ 1.73) in the NOESY spectrum. Therefore, we determined the new compound as 7-(((2′Z,6′Z)-5′,9′-hydroxy-3′,7′-dimethylocta-2′,6′-dien-1′-yl)oxy)-2*H*-chromen-2-one and we named it as 2′-Z auraptene D.

### The proposed transformation pathway

**Fig. 3:**
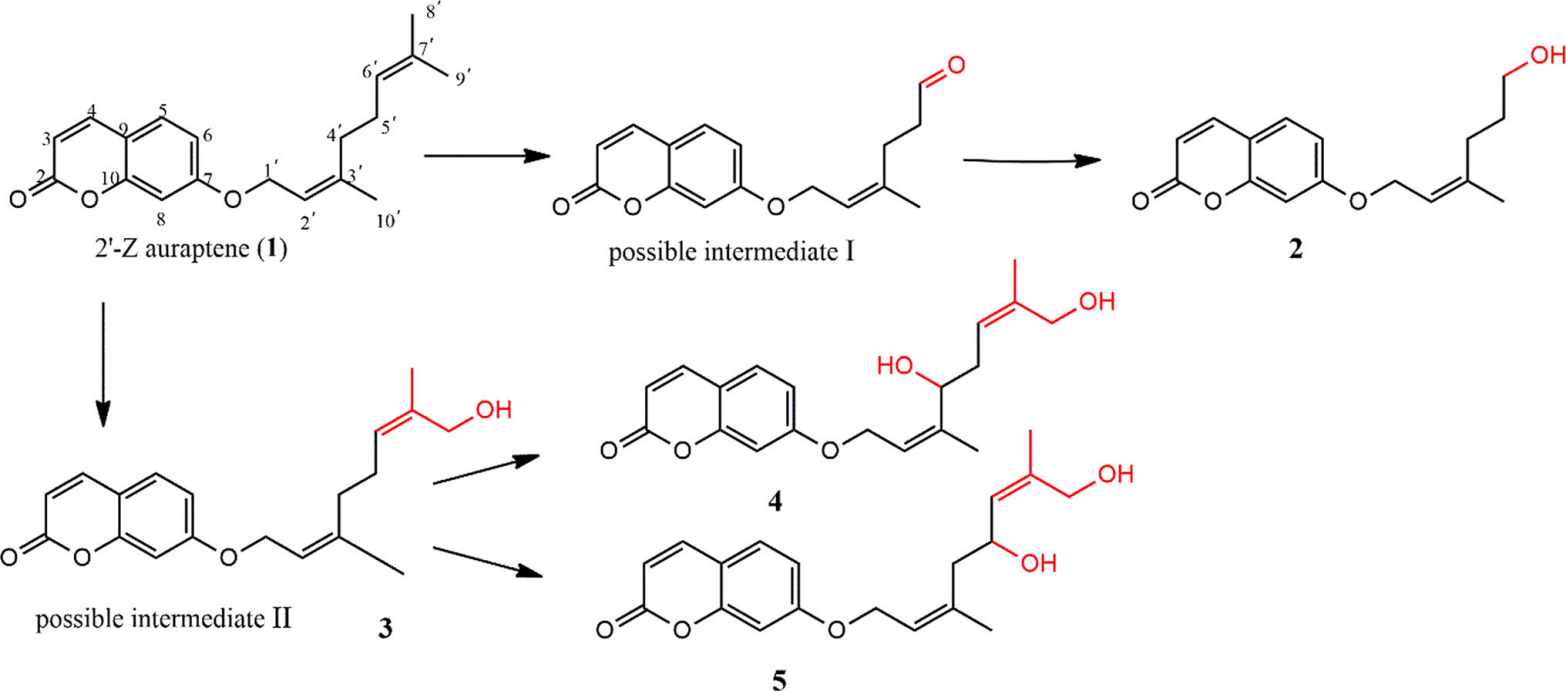
The proposed transformation pathway of compounds **2**–**5**

A plausible biotransformation pathway of *cis*-auraptene by *Mucor polymorphosporus* was proposed as shown in Fig. 3. The formation of the possible intermediate I could be initiated by the epoxidation reaction of olefinic bond at C-6′-C-7′, followed by the break of C–C bond to form a carbonyl group at C-6′. And then the carbonyl group was reduction to obtain the compound **2** (Sayed 2001). Besides, the possible intermediate II, 2′-Z auraptene B (**3**), was formed through the hydroxylation of C-9′, which was further hydroxylated at C-4′ or C-5′ to obtain compound **4** or compound **5**, respectively (Silva et al. 2013; Niu et al. 2008). The biotransformation of *cis*-auraptene by *Mucor polymorphosporus* involved oxidation, hydroxylation and reduction. It is obviously that the hydroxylation occurs mainly at C-9′, which is a selective reaction of *Mucor polymorphosporus.* This indicates a new method to selectively hydroxylation for the methyl in *cis*-auraptene, which was difficult to achieve by common synthetic methods.

### 2′-Z auraptene A (**2**) preferentially inhibits the cell growth of human gastric cancer MGC-803 cells

**Fig. 4:**
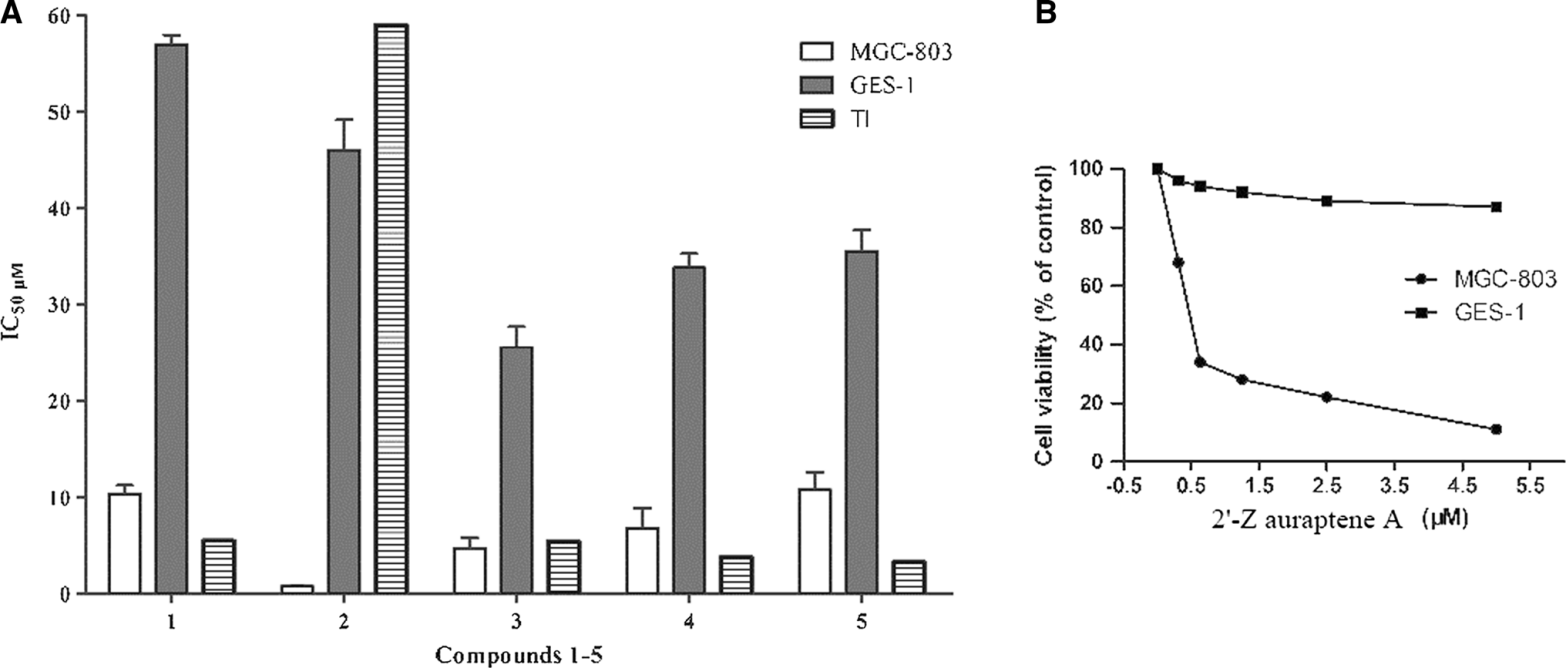
The antiproliferative effect of compounds **1**–**5** against MGC-803 and GES-1 cells; TI values represent IC_50_ (GES-1)/IC_50_ (MGC-803))

We first researched the antiproliferative effect of 2′-Z auraptene (**1**) and four new metabolites (**2**–**5**) obtained from the biotransformation of **1** by *Mucor polymorphosporus* at human gastric cancer MGC-803 cells and human epithelial cell line GES-1, as shown in Fig. 4 A (Supplementary Data). Compound **2** showed preferential pharmacological activities with an IC_50_ value of 0.78 ± 0.13 μM and therapeutic index (TI) value of 59.0, which are significantly improved from that of 2′-Z auraptene (IC_50_ = 10.38 ± 0.86 μM, TI = 5.5), which indicated that 2′-Z auraptene A (**2**) was the most potent against MGC-803 gastric cancer cells and less cytotoxic against GES-1 cell line compared with MGC-803 cells (Fig. 4B). Therefore, compound **2** was chosen for further study.

### 2′-Z auraptene A (**2**) induced apoptosis in MGC-803 cells

**Fig. 5:**
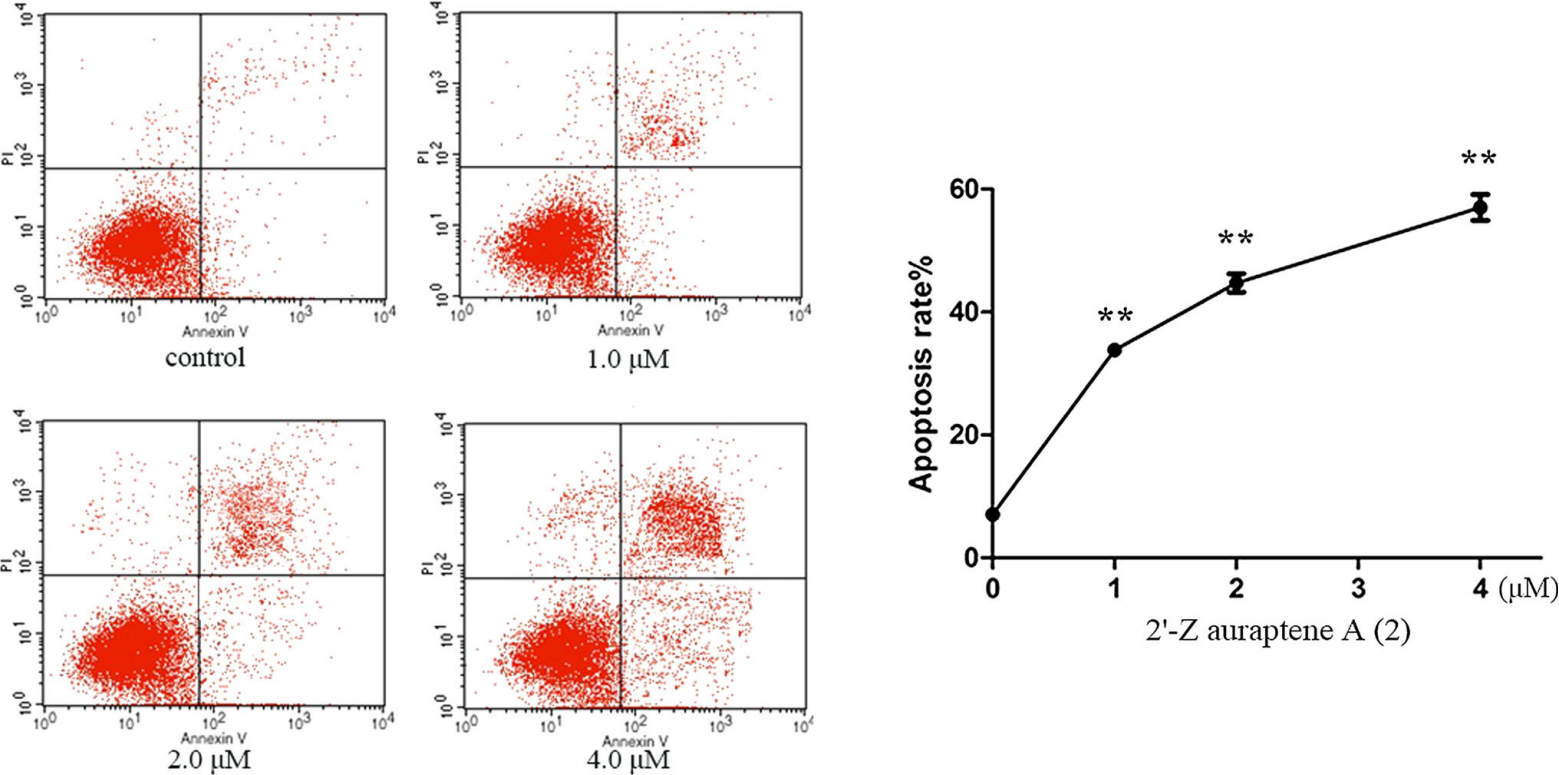
Compound **2** induced apoptosis in MGC-803 cells detected by annexin V-FITC/PI staining test. **p < 0.01 were considered statistically significance

**Fig. 6:**
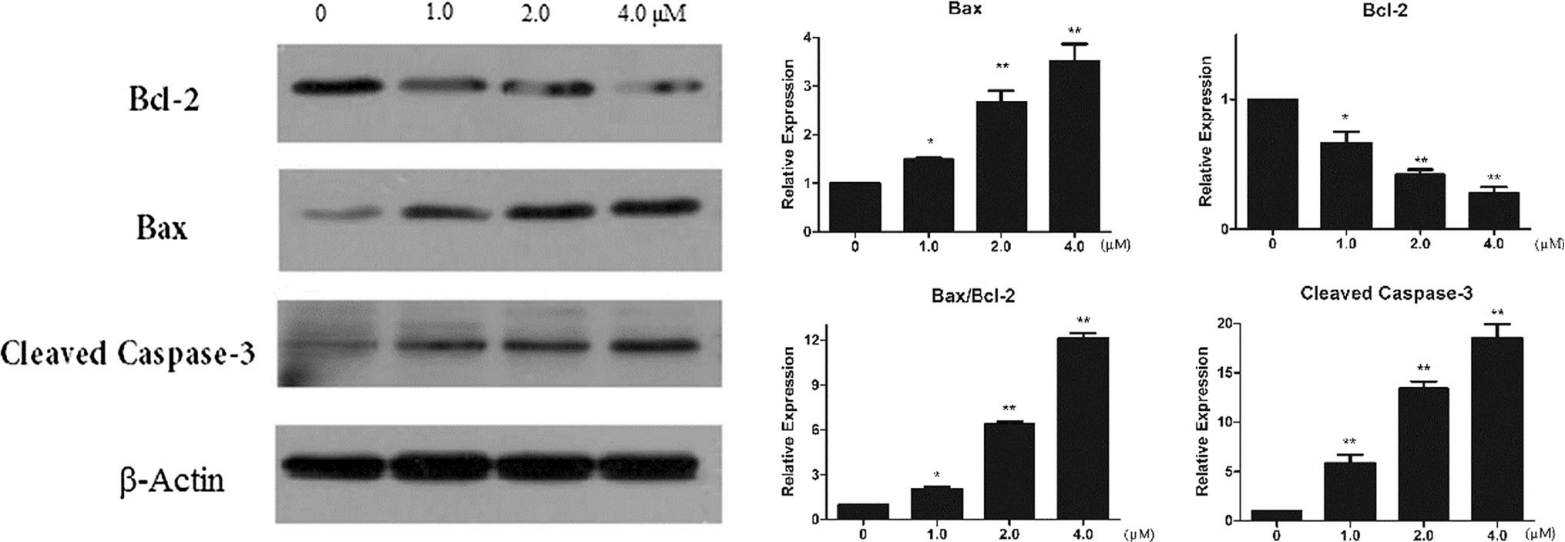
The effects of compound on the expression of apoptosis-related proteins determined by western blot. *p < 0.05 and **p < 0.01 were considered statistically significance

To established the effect of 2′-Z auraptene A (**2**) on inducing apoptosis, MGC-803 cells were treated with compound **2** for 24 h and submitted to flow cytometry analyses. The celluar apoptotic rates in compound **2**-treated groups increased significantly in a dose-dependent manner compared with the control group (Fig. 5). Besides, we analysed the protein expression of Bax, Bcl-2 and cleaved caspase-3 after treatment with compound **2**. The results exhibited a reduction in the level of Bcl-2, an increase of Bax and an increase in cleaved caspase-3 protein after treating with compound **2** (Fig. 6). Relative protein expression results are also shown in Fig. 6. Therefore, these results indicated that 2′-Z auraptene A could inhibited MGC-803 gastric cancer cells growth through inducing dose-dependent apoptosis.

## Discussion

In the present study, biotransformation by *Mucor polymorphosporus* was used to modify the structure of *cis*-auraptene and its high selectivity of hydroxylation reaction at C-9′ showed much advantage compared with the traditional chemical methods (Hoekstra 2001). Besides, the results of biotransformation also indicated that *Mucor polymorphosporus* was specific at the hydroxylation reaction, which could be a new solution for selectively hydroxylation for the methyl of *cis*-olefinic bond. Based on numerous studies of active coumarins, 7-hydroxycoumarins have been described as cancer chemoprevention, but only few reports of them on human gastric cancer cell line and no reports on the biotransformation of active coumarins. In our study, four new coumarins were obtained from biotransformation by *Mucor polymorphosporus,* and compound **2** exhibited the best anticancer activity against MGC-803 cells with higher therapeutic index, indicating that *cis*-auraptene A could be a promising lead compound with efficacy and specificity against human gastric cancer cell line. Our studies suggested that compound **2** induces dose-dependent apoptosis indicating the possible mechanism for its antiproliferative activity against gastric cancer cells.

In conclusion, the synthetic active compound, 2′-Z auraptene, was modify through biotransformation, and 2′-Z auraptene A was the most effective and specific compound in the antiproliferative effects against gastric cancer MGC-803 cells among the four new metabolites. Besides, compared with 2′-Z auraptene, the limitation of solubility of compound **2** in aqueous media under thermodynamic equilibrating conditions was improved and it could also induce significantly apoptosis effect. Therefore, 2′-Z auraptene A could be described as promising candidate for further anti-tumor drug development.

## Electronic supplementary material

Below is the link to the electronic supplementary material.

Supplementary material 1 (PDF 1305 kb)
