# Supplementary material for: A new terpene coumarin microbial transformed by Mucor polymorphosporus induces apoptosis of human gastric cancer cell line MGC-803
Source: Arch Pharm Res. 2018 Apr 4;41(6):646–54. doi: 10.1007/s12272-018-1028-0 (PMC6028838; doi:10.1007/s12272-018-1028-0)
Supplement: Supplementary file 1 — Supplementary material 1 (PDF 1305 kb) [file 12272_2018_1028_MOESM1_ESM.pdf]

Supplementary Data

A new terpene coumarin microbial transformed by Mucor polymorphosporus induces apoptosis of human gastric cancer cell line MGC-803

Guangzhi Li[1, 2]•Junchi Wang[1]•Xiaojin Li[3]•Jianguo Xu[3]•Zhao Zhang[1] *•Jianyong Si[1] *

1. Key Laboratory of Bioactive Substances and Resources Utilization of Chinese Herbal Medicine, Ministry of Education, Institute of Medicinal Plant Development, Chinese Academy of Medical Sciences, Peking Union Medical College, Beijing 100193, China;

2. Shenzhen Luohu People's Hospital, The Third Affiliated Hospital of Shenzhen University, Shenzhen 518035, PR China;

3. Xinjiang Institute of Chinese Materia Medica and Ethical Materia Medica, Urumqi 830002, China

* Corresponding authors. Tel. /Fax: +86-010-57833299.
E-mail: zzhang@implad.ac.cn； jysi@implad.ac.cn；

MS and NMR Spectra of Compounds

Figure 1. HR-ESI-MS spectrum of 2

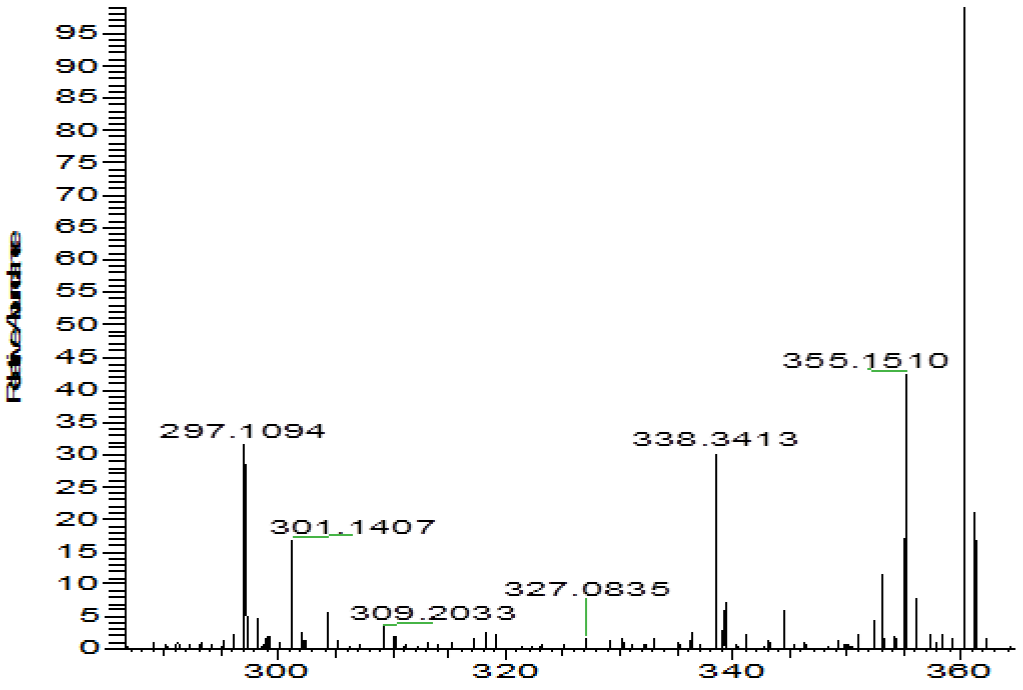

Figure 2. ^{1}H NMR spectrum of 2

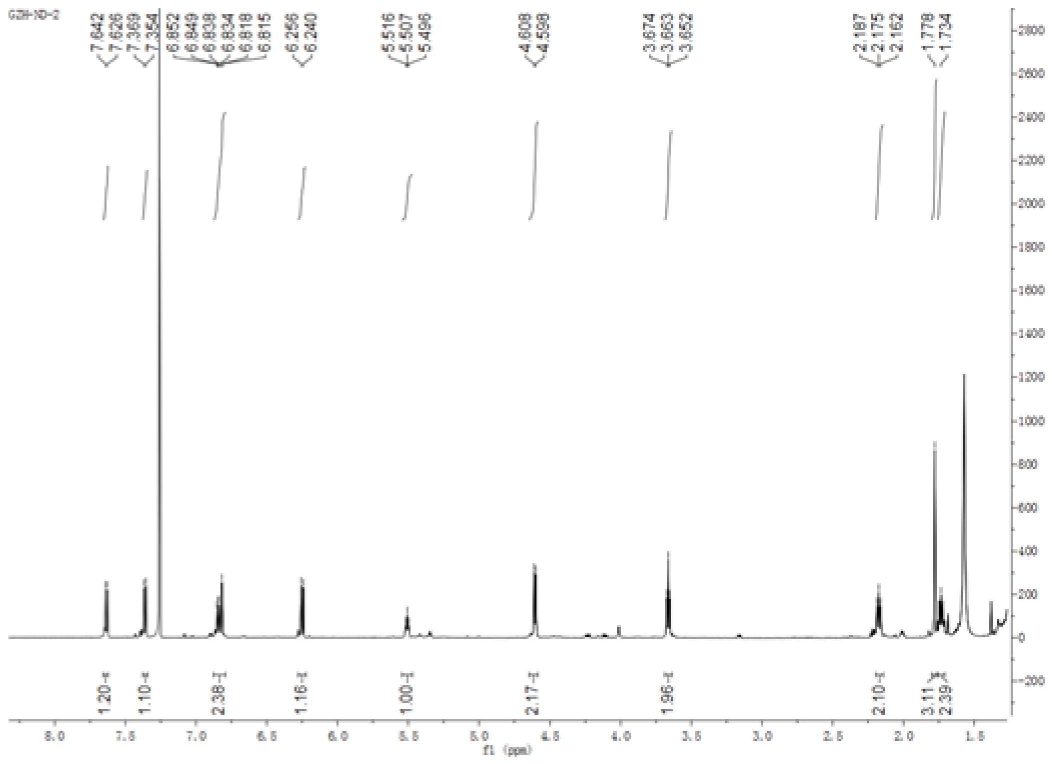

Figure 3. ^{13}C NMR spectrum of 2

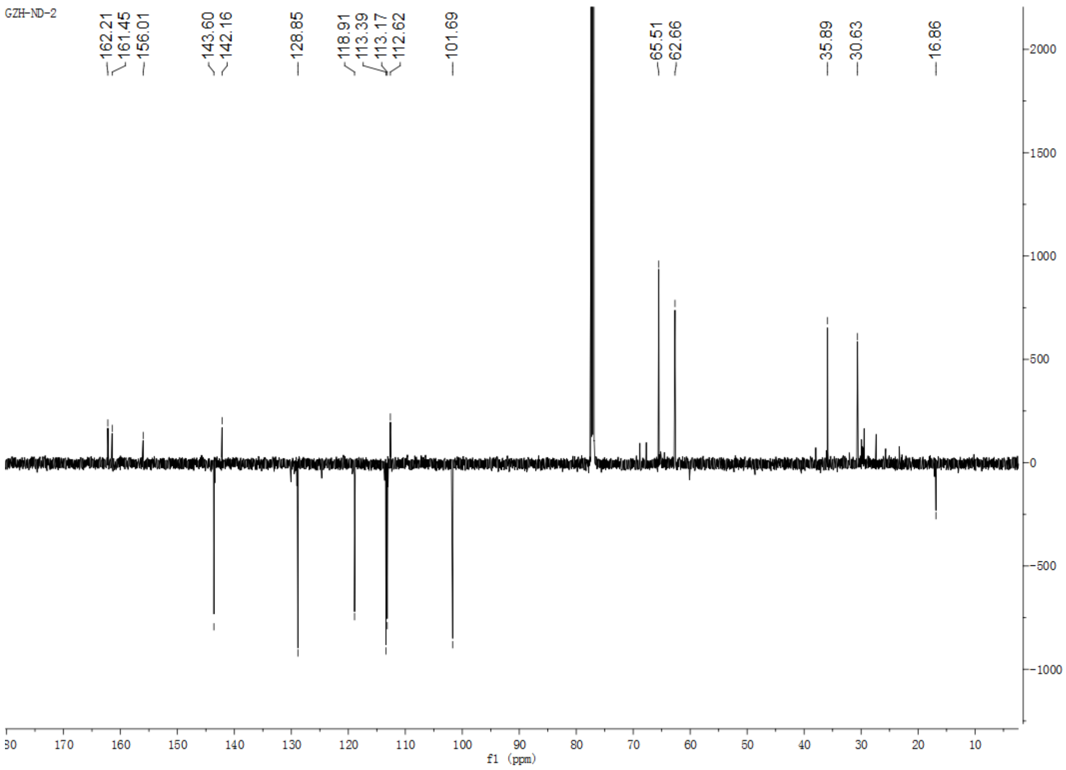

Figure 4. HMBC spectrum of 2

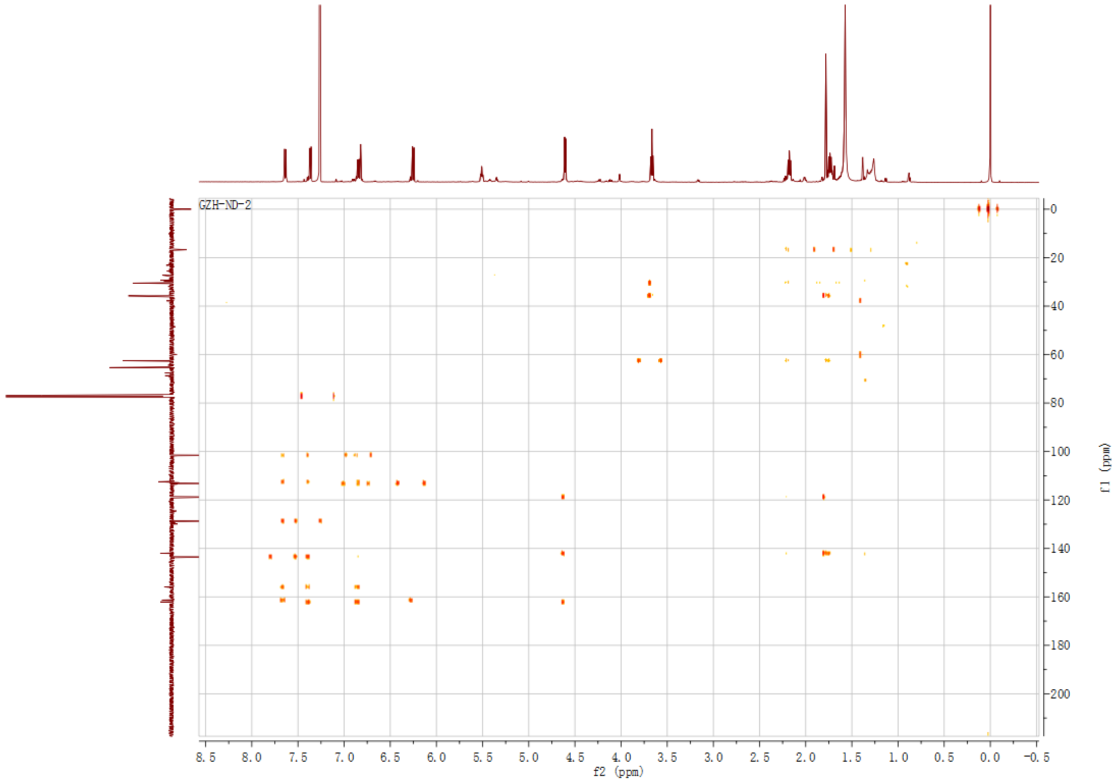

Figure 5. HR-ESI-MS spectrum of 3

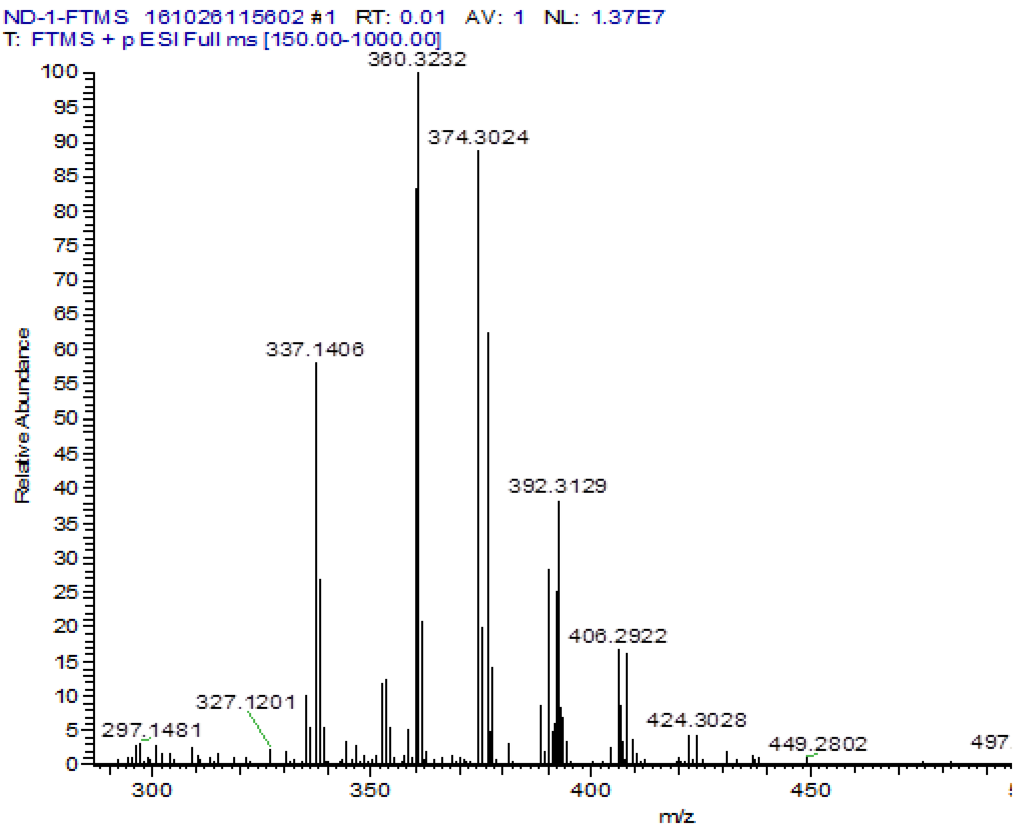

Figure 6. ^{1}H NMR spectrum of 3

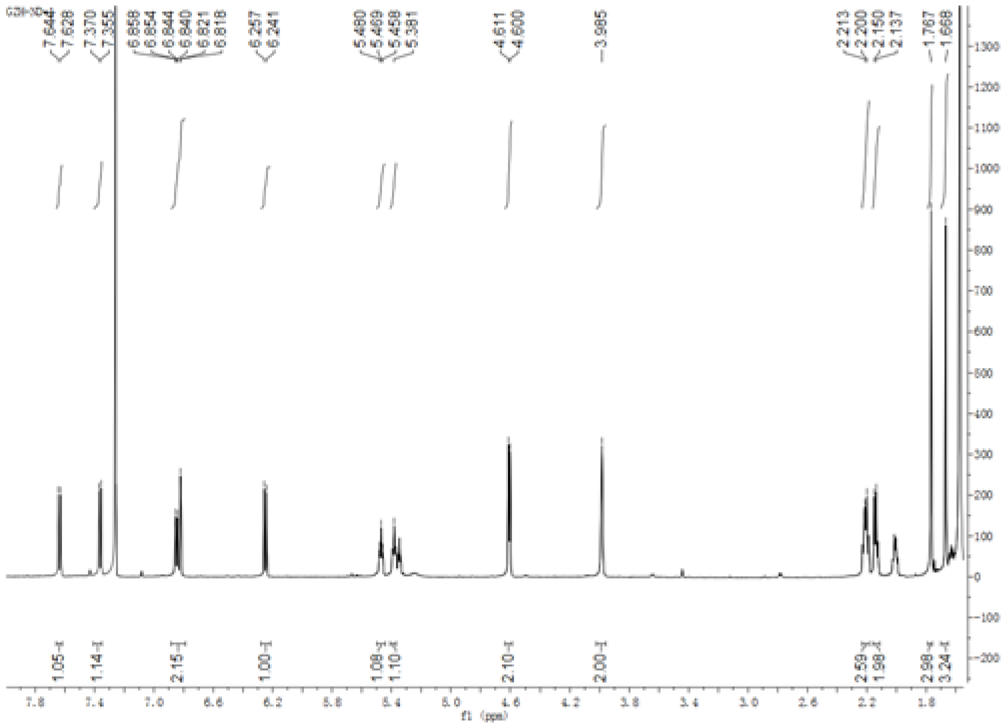

Figure 7. ^{13}C **NMR spectrum of 3**

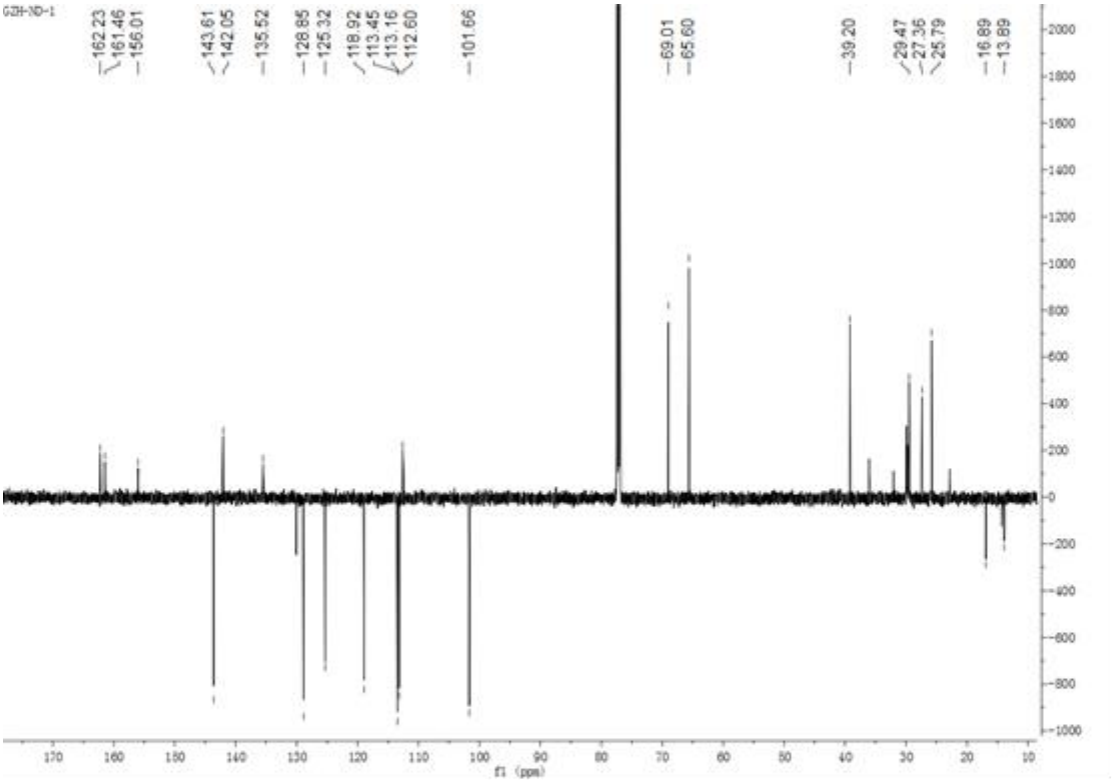

Figure 8. HMBC spectrum of 3

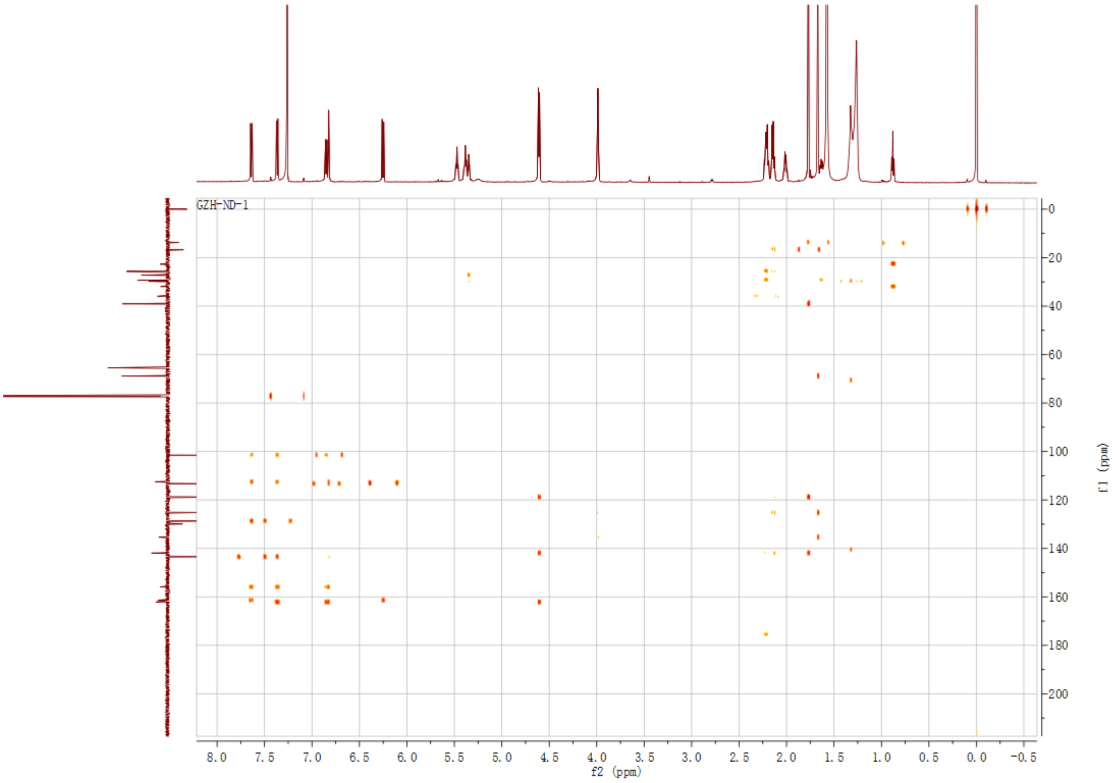

Figure 9. NOESY spectrum of 3

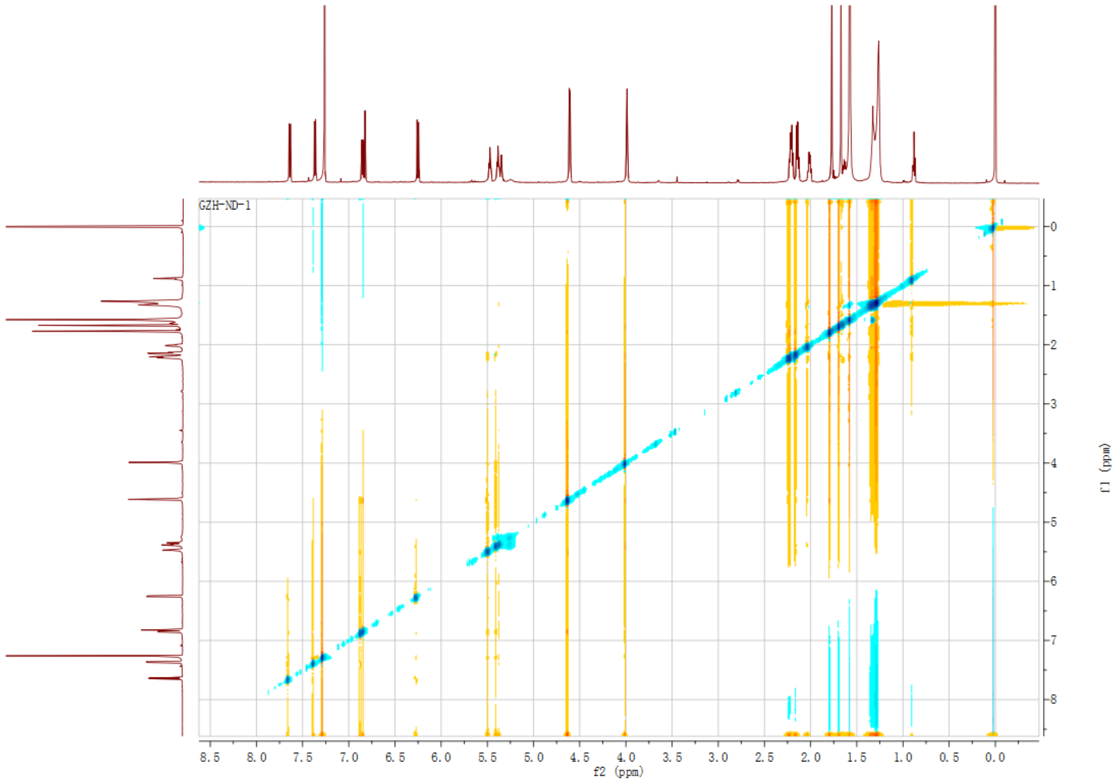

Figure 10. HR-ESI-MS spectrum of 4

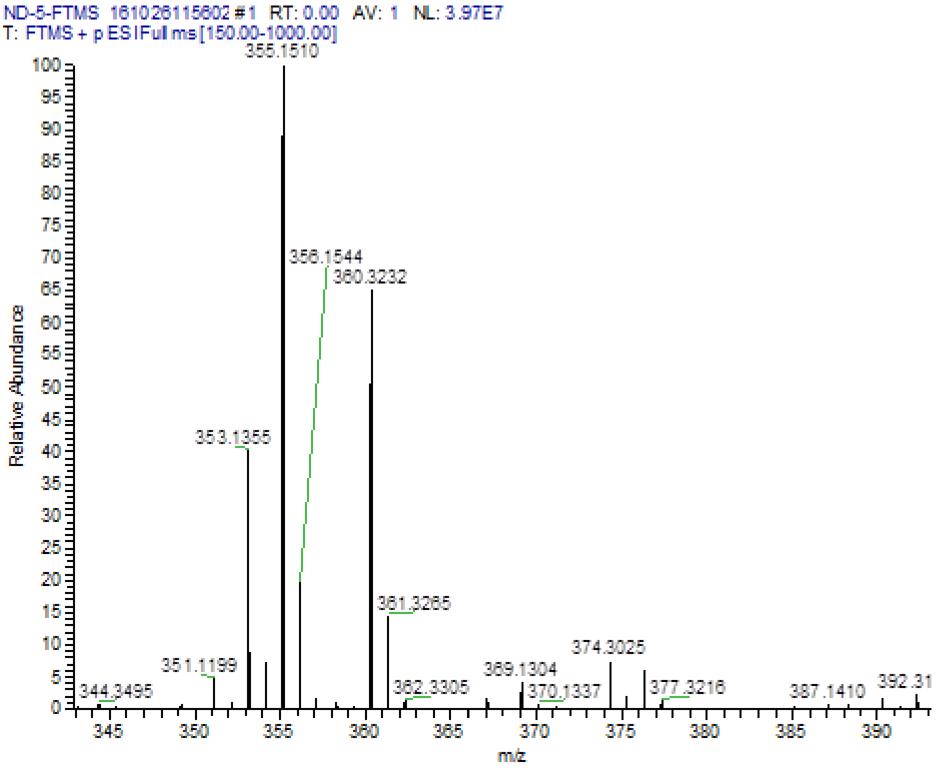

Figure 11. ^{1}H NMR spectrum of 4

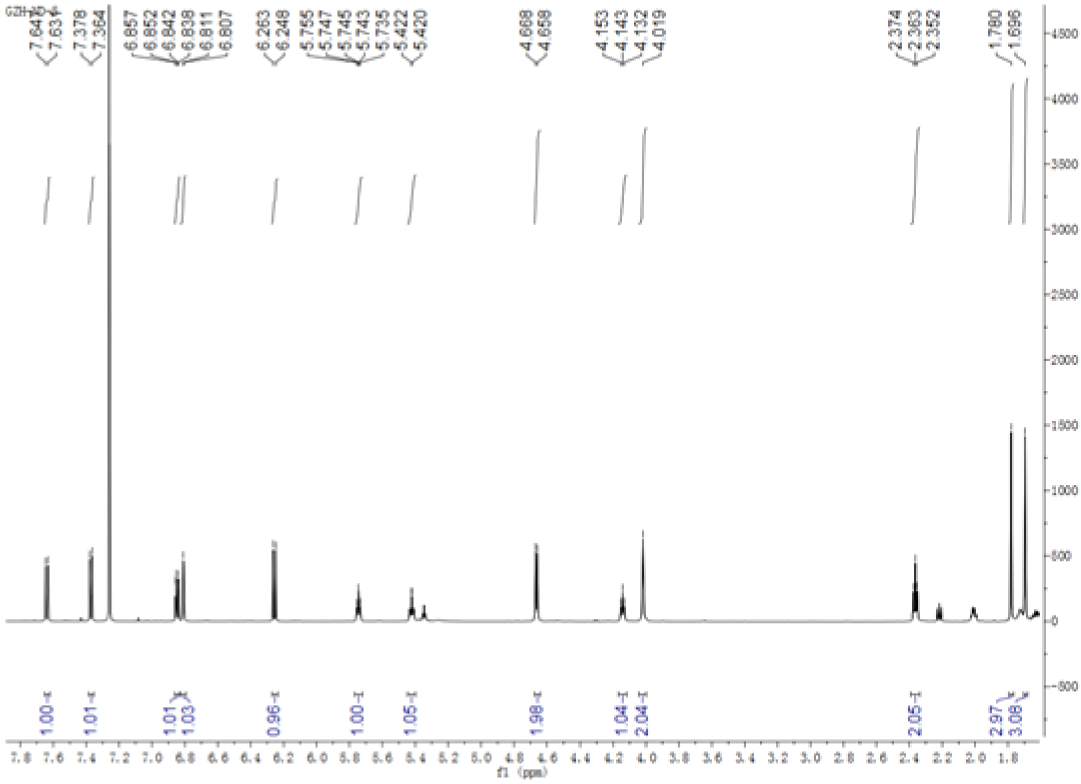

Figure 12. ^{13}C NMR spectrum of 4

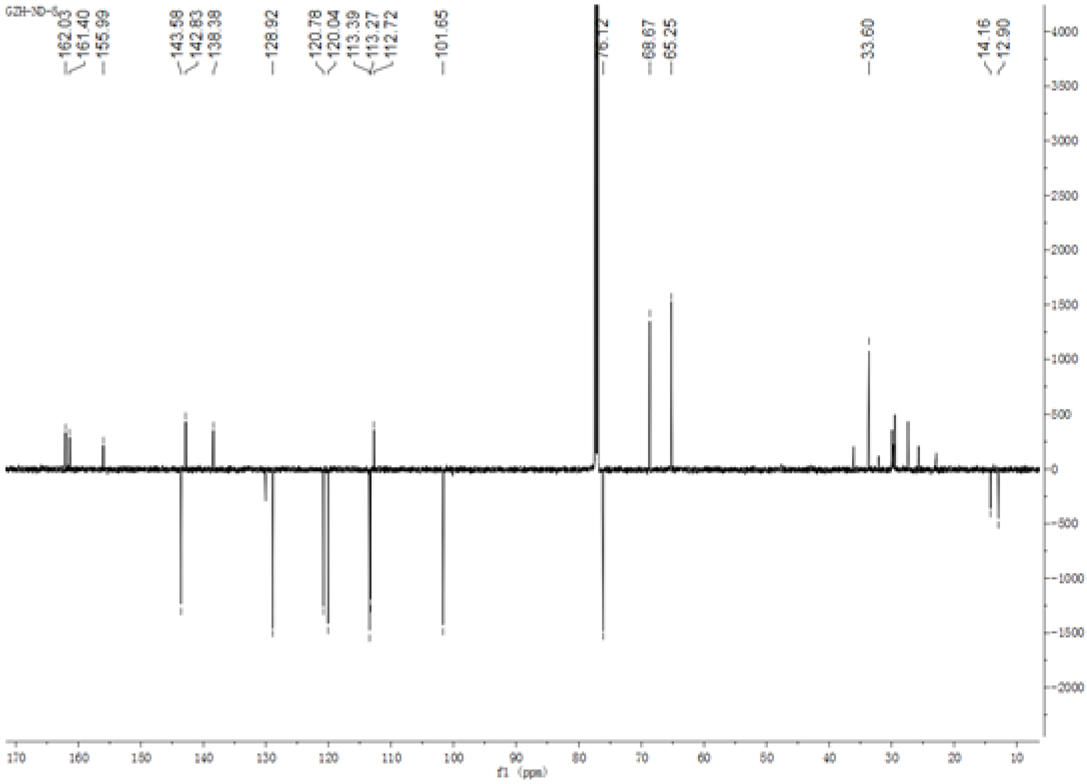

Figure 13. HMBC spectrum of 4

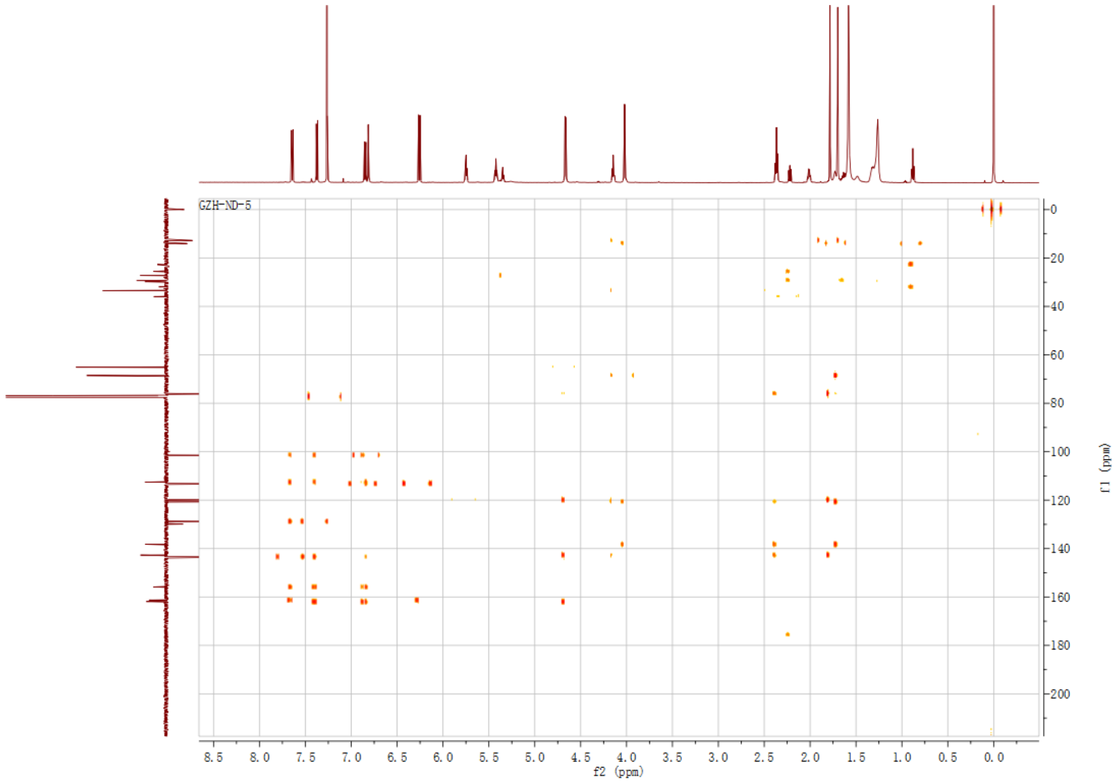

Figure 14. NOESY spectrum of 4

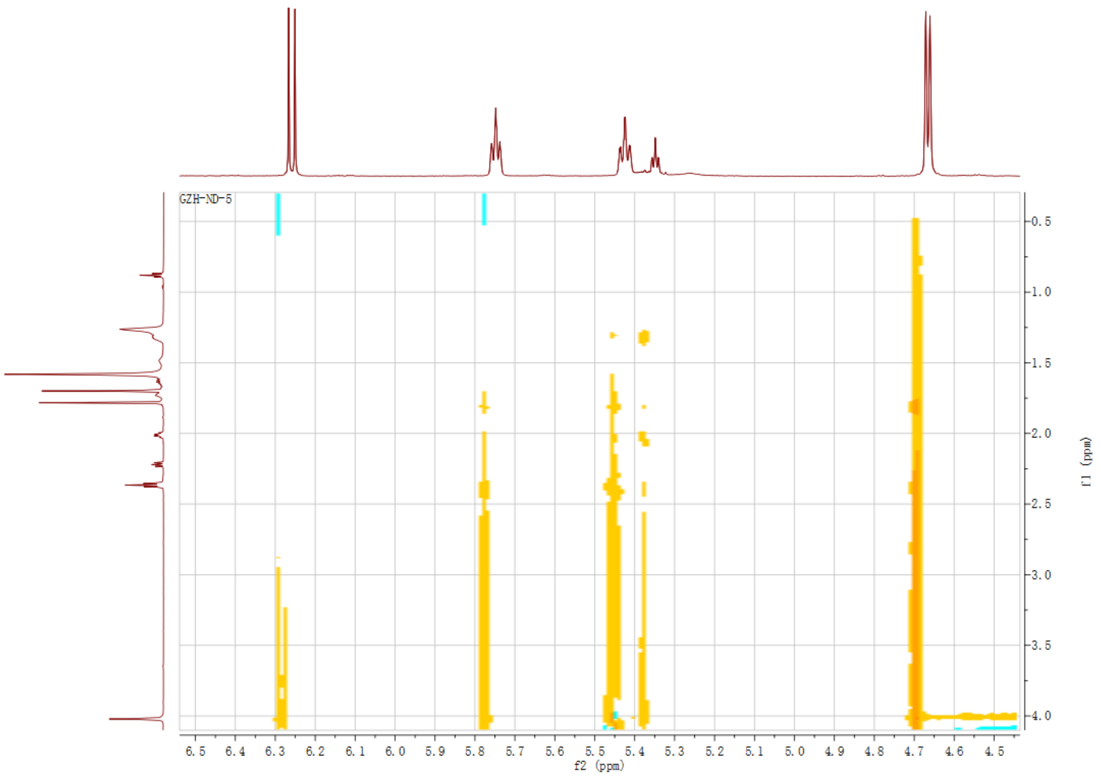

Figure 15. HR-ESI-MS spectrum of 5

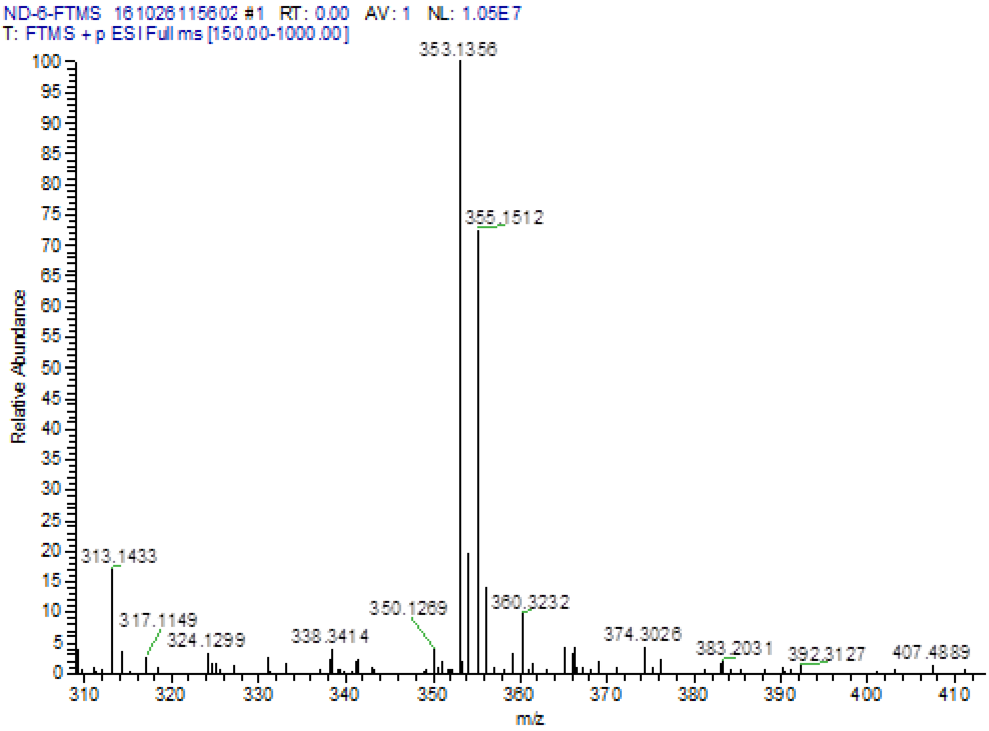

Figure 16. ^{1}H NMR spectrum of 5

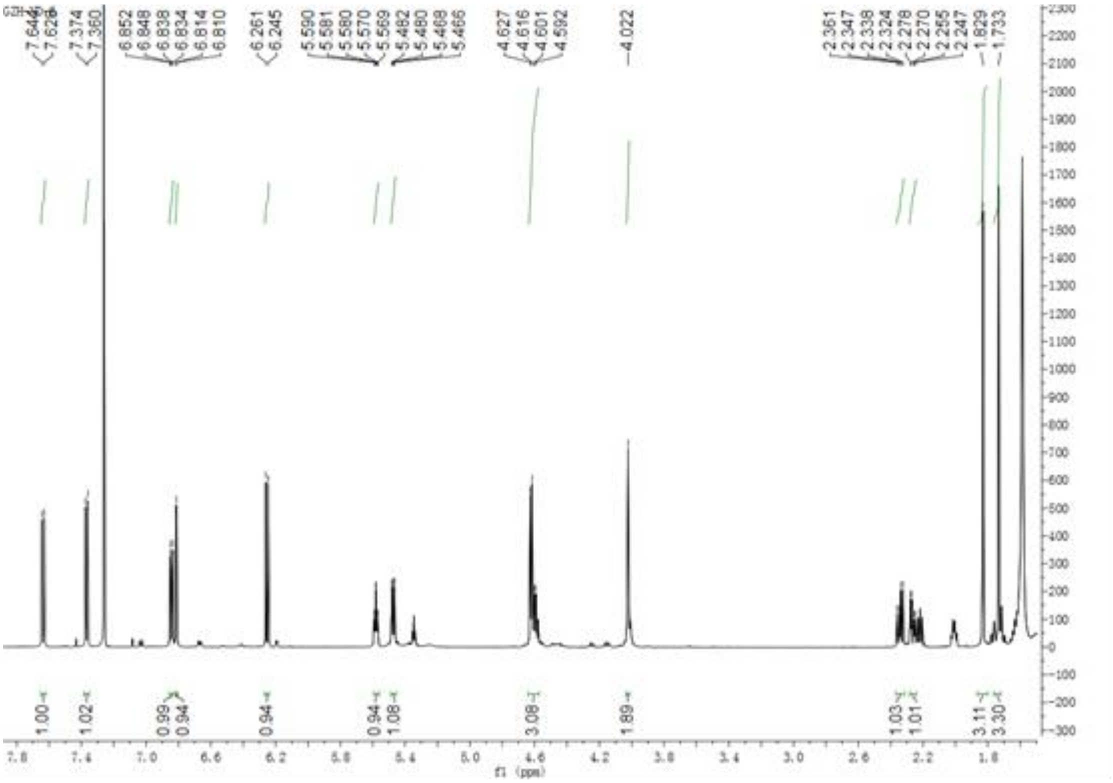

Figure 17. ^{13}C NMR spectrum of 5

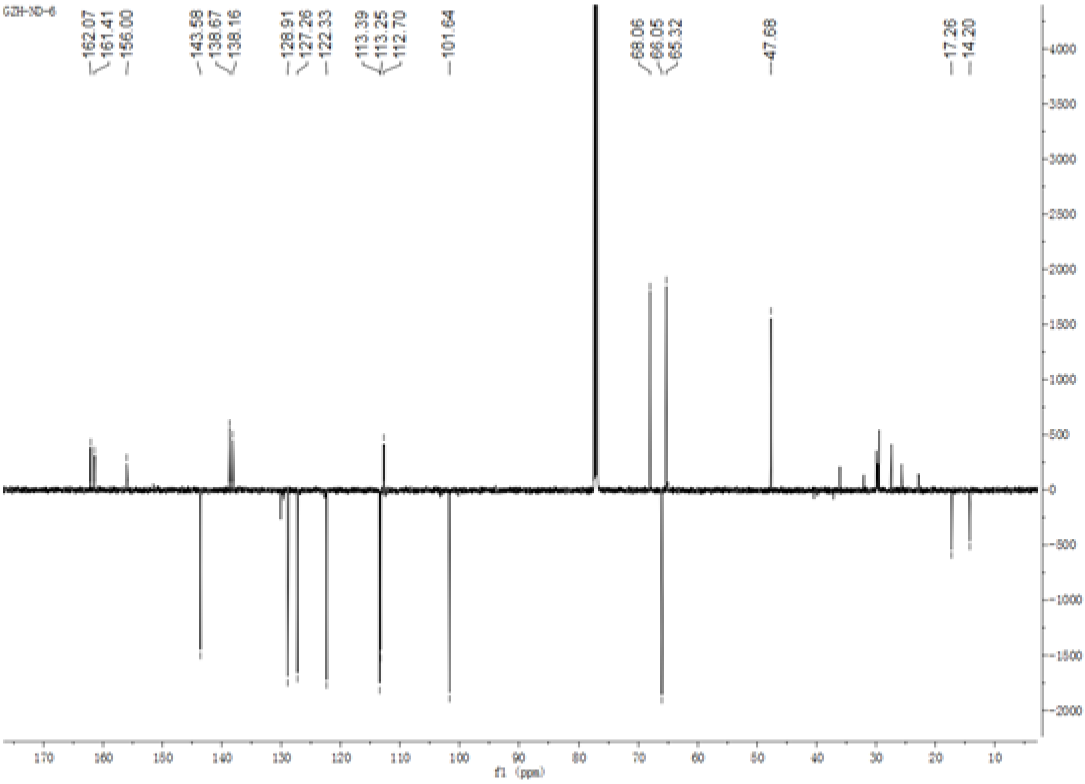

Figure 18. NOESY spectrum of 5

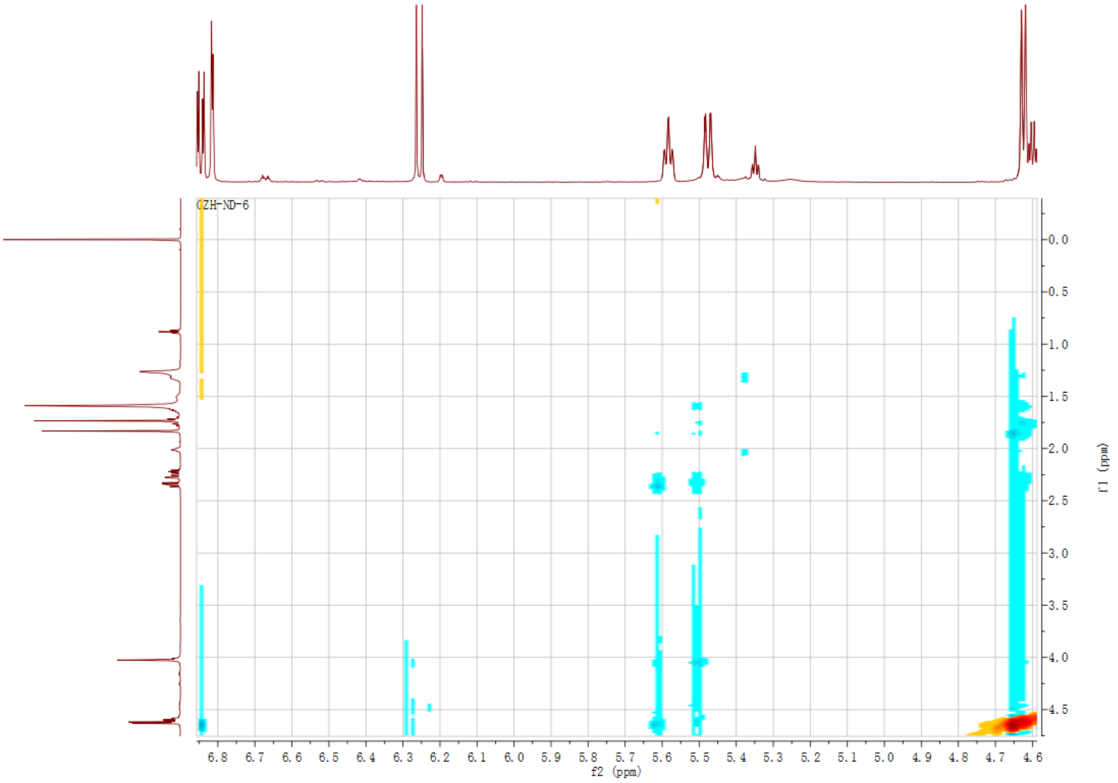

Figure 19. HMBC spectrum of 5

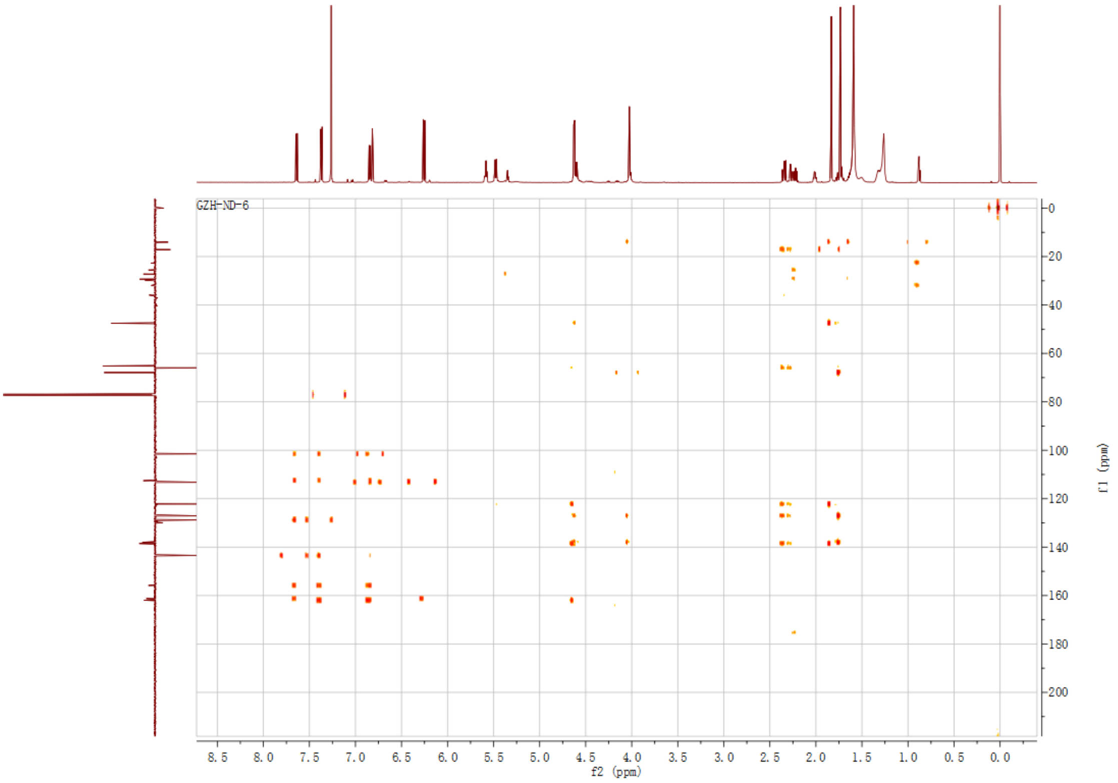

Table . Cytotoxicity of compounds **1-5**

Compound	IC_{50} (μM)		TI
	MGC-803	GES-1	
1	10.38 ± 0.86[a]	56.96± 2.31	5.5
2	0.78 ± 0.13	46.04 ± 3.11	59.0
3	4.75 ± 1.02	25.56± 2.12	5.4
4	6.78 ± 2.06	33.82 ± 1.46	3.8
5	10.78 ± 1.83	35.5 ± 2.21	3.3

IC_{50} represent the concentration of compound causing 50% growth inhibition of cells; a Values represent mean ± SD of triplicate experiments; TI values represent IC_{50} (GES-1)/ IC_{50} (MGC-803)
